# Thermal Change and the Dynamics of Multi-Host Parasite Life Cycles in Aquatic Ecosystems

**DOI:** 10.1093/icb/icw025

**Published:** 2016-06-01

**Authors:** Iain Barber, Boris W. Berkhout, Zalina Ismail

**Affiliations:** Department of Neuroscience, Psychology & Behaviour, College of Medicine, Biological Sciences and Psychology, University of Leicester, Leicester, LE1 7RH, UK

## Abstract

Altered thermal regimes associated with climate change are impacting significantly on the physical, chemical, and biological characteristics of the Earth’s natural ecosystems, with important implications for the biology of aquatic organisms. As well as impacting the biology of individual species, changing thermal regimes have the capacity to mediate ecological interactions between species, and the potential for climate change to impact host–parasite interactions in aquatic ecosystems is now well recognized. Predicting what will happen to the prevalence and intensity of infection of parasites with multiple hosts in their life cycles is especially challenging because the addition of each additional host dramatically increases the potential permutations of response. In this short review, we provide an overview of the diverse routes by which altered thermal regimes can impact the dynamics of multi-host parasite life cycles in aquatic ecosystems. In addition, we examine how experimentally amenable host–parasite systems are being used to determine the consequences of changing environmental temperatures for these different types of mechanism. Our overarching aim is to examine the potential of changing thermal regimes to alter not only the biology of hosts and parasites, but also the biology of interactions between hosts and parasites. We also hope to illustrate the complexity that is likely to be involved in making predictions about the dynamics of infection by multi-host parasites in thermally challenged aquatic ecosystems.

## Introduction

Altered thermal regimes associated with climate change are impacting significantly on the physical, chemical, and biological characteristics of the Earth’s natural ecosystems (IPCC 2013), with important implications for the biology of aquatic organisms (e.g., Pörtner and Knust 2007). Over the next century, increases in both the mean temperature and the incidence of extreme temperature events are predicted (Meehl and Tebaldi 2004; IPCC 2013). As well as impacting the biology of individual species, changing thermal regimes have the capacity to mediate ecological interactions between species, and the potential for climate change to impact host–parasite interactions in aquatic ecosystems has long been recognized (Marcogliese 2001; Lafferty 2009).

Thermal changes arising from climate change are often expected to favor parasites over hosts, through expanded host or parasite ranges and/or reduced immunocompetence of hosts, and hence to lead to more severe effects of parasites and disease under future climate scenarios (Patz et al. 2003; Purse et al. 2005; Pounds et al. 2006). However, while some parasites will undoubtedly benefit from altered climate scenarios, there is an increasing recognition of the diversity of possible responses, with many parasites likely to experience barriers to transmission under new environmental regimes, as host populations are impacted and conditions for transmission are altered (Lafferty 2009; Lõhmus and Björklund 2015). Parasites have their own geographic limits that are constrained not only by host ranges but also by the physicochemical demands of free-living transmission stages (Bozick and Real 2015), which are in direct contact with the aquatic environment and hence are susceptible to thermal or other perturbations (Vidal-Martínez et al. 2015). Furthermore, parasites that are transmitted through aquatic food webs not only require the presence of healthy populations of multiple host species, but also rely on ecological (i.e., predator–prey) interactions between these species for transmission to be fulfilled, with either (or both) of these being impacted under changing environments (Lafferty 2009). Therefore, more pristine ecosystems are expected to be characterized by richer parasite diversity than degraded ecosystems (Hudson et al. 2006).

**Fig. 1:**
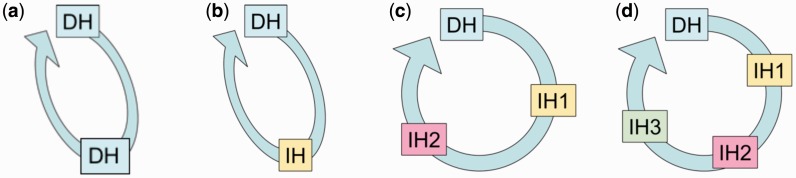
The types of parasite life cycles encountered in aquatic ecosystems vary in complexity from simple direct life cycles (a) in which transmission occurs between two definitive hosts (DH) of the same species, to more complex, indirect life cycles that involve transmission of parasites between multiple obligate intermediate hosts (IH) of different species (b–d). (This figure is available in black and white in print and in color at *Integrative And Comparative Biology* online).

Predicting what will happen to the prevalence and intensity of infection of parasites with multiple hosts in their life cycles is especially challenging, since the addition of each additional host dramatically increases the potential permutations of response (Fig. 1). A long history of experimental studies examining how environmental factors alter the development, survival, behavior, and infectivity of developmental stages of parasite infections suggests that temperatures are likely to influence many aspects of host–parasite interactions. However, to develop a more predictive understanding of how patterns of infection will change under altered climates there is a need to develop a greater understanding of how temperature influences parasites at all stages of their life cycles, as well as their interactions with all hosts. One reason for the difficulty of the task is that changing temperatures affect not only the biology of the parasite and host taxa involved, but also the interactions between them. For parasites whose life cycles demand that multiple obligate host species are exploited in a particular sequence, the effects of temperature on multiple hosts, multiple parasite stages, and multiple host–parasite interactions need to be considered to understand the implications for life cycle dynamics. As the complexity of life cycles increases, so does the level of the challenge involved in solving these puzzles (Fig. 1a–d).

## Aims of the review

In this article, we provide an overview of the diversity of routes by which altered thermal regimes can impact the dynamics of multi-host parasite life cycles in aquatic ecosystems. In addition, we examine how experimentally amenable host–parasite systems are being used to determine the consequences of environmental temperature change for each of these types of mechanism. Our article does not aim to provide a comprehensive treatise on all possible implications of climate change for host–parasite interactions. Instead, our overarching aim is to examine how thermal change has the potential to alter not only the biology of hosts and parasites, but also the biology of interactions between hosts and parasites. We hope this approach will develop an appreciation of the complexity that is likely to be involved in making predictions about the dynamics of infection by multi-host parasites in thermally challenged aquatic ecosystems.

## Implications of changing thermal environments for host–parasite interactions in aquatic ecosystems

**Fig. 2:**
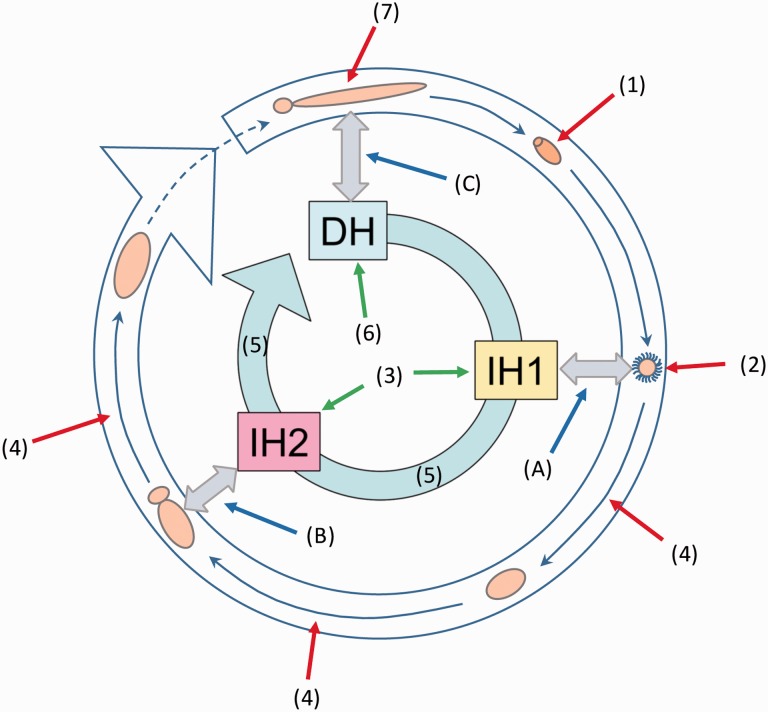
Here we use the life cycle of a three-host, indirectly transmitted, parasite as a framework to graphically summarize the various mechanisms by which altered thermal regimes can influence the dynamics of multi-host parasite life cycles in aquatic ecosystems. Inner circle shows hosts, outer circle shows parasite stages. Numbers relate to manuscript sections. (1) Effects on the survival and development of embryonic parasite stages; (2) effects on the survival, activity, and host finding ability of free-swimming infective stages; (3) effects on the susceptibility of intermediate hosts to infective parasites; (4) effects on the survival, growth, and development of parasite stages in intermediate hosts; (5) effects on the ecology and behavior of intermediate hosts; (6) effects on the biology and ecology of definitive hosts; and (7) effects on the performance of adult parasites infecting definitive hosts. Effects of temperature on the interactions between infective parasites and prospective hosts (“A,” “B,” and “C”) are also shown.

In this section, we follow the framework presented in Fig. 2 to examine how altered thermal regimes associated with climate change influence host–parasite interactions throughout a generalized multi-host life cycle.

### Survival and development of embryonic parasite stages

For almost all endoparasites that have multi-host life cycles, eggs produced by the adult parasite are released with the faeces of definitive hosts into the external environment (Smyth 1994; Goater et al. 2014). While some parasites achieve transmission through passive mechanisms, with static eggs being consumed by intermediate hosts (e.g., acanthocephalans; Cheng 1973), in many cases eggs hatch to release free-swimming infective stages, which typically exhibit behavioral adaptations to facilitate transmission (e.g., cestode coracida, trematode miracidia; Cheng 1973; Dubinina 1980). Following deposition into the external environment, egg-bound embryos normally require a period of development before they are ready to hatch or otherwise be competent to be transmitted to the (first) intermediate host, and this development is sensitive to environmental temperature. Since elevated temperatures typically increase the metabolic rate of ectothermic organisms (Schmidt-Nielsen 1997), an increase in parasite development rate—and hence a decrease in the embryonation period—in warmed environments is often reported (Sakanari and Moser 1985; Measures 1996; Paull and Johnson 2011). However, while embryonation periods can be sharply reduced with increasing temperature, eggs are non-feeding (“lecithotrophic”) stages and thus possess limited energy reserves, which may be depleted faster with increased metabolism. Consequently, warmer temperatures have the potential to decrease the survival probability and hence persistence of eggs following embryonation (Nollen et al. 1979). As a result, temperature effects on egg hatching are often nonlinear, with intermediate temperatures typically generating the highest overall hatching success. The temporal consistency of future thermal regimes will also likely have a major influence on egg development times. While predicted increases in the frequency of extreme temperature events might reduce the development time of embryonic parasites if they increase thermal stochasticity (Saunders et al. 2002), they could have negative impacts if critical thresholds are exceeded.

### Survival, activity, and host finding ability of free-swimming infective stages

Free-living, motile infective parasite stages, emerging from embryonated eggs or from intermediate hosts following processes of asexual multiplication, must locate and invade susceptible subsequent hosts either by penetrating the gut wall following ingestion (e.g., as in cestodes and some nematodes; Cheng 1973; Dubinina 1980), or by penetrating the external host tegument from the environment (e.g., trematodes and some nematodes; Cheng 1973). Assuming that susceptible intermediate hosts are available under altered thermal regimes (section “Susceptibility of intermediate hosts to infective parasites”), the longevity and activity levels of infective free-living parasite stages—as well as their habitat selection and ability to use effective host-finding behaviors—is likely to influence the probability of host encounters. Each of these processes is potentially affected by temperature changes.

Free-swimming stages, like egg-bound embryos, are lecithotrophic and contain a finite amount of energy to sustain life and fuel locomotion. Under higher temperatures their activity levels typically increase (Koprivnikar et al. 2010) and consequently these resources are consumed more rapidly (Pechenik and Fried 1995). A number of studies have documented the reduced longevity of such stages with increasing temperature, including the miracidia and cercariae of trematodes and the coracidia of cestodes (Nollen et al. 1979; Pietrock and Marcogliese 2003; Koprivnikar et al. 2010), although others show a dome-shaped response (Lo and Lee 1996). However, most species appear to have the capacity to tolerate temperature ranges within those commonly encountered under natural field conditions, and there appears to be potential for the increased activity and infectivity of cercariae at higher temperatures to compensate for their lower survival time (Evans 1985; McCarthy 1999, Morley 2011). Hence, it is unclear to what extent the survival and activity of free-swimming parasite stages will be affected by moderate temperature changes (Measures 1996; Morley 2012), though extreme temperatures associated with heat waves may impact severely.

Other mechanisms, however, might be expected to impact these stages following relatively small, incremental rises in temperature. Motile, free-living infective stages of heteroxenous aquatic parasites, including trematode miracidia and cercariae, have evolved specific behaviors that facilitate the effective location of susceptible hosts (Haas and Haberl 1997). These include simple tropic and tactic responses—including phototropism (Haas et al. 2008) and chemotaxis (Haas et al. 1995)—that are potentially susceptible to temperature. However, the extent to which these abilities will be affected by changing temperatures is still unclear (Lee et al. 2013). Recent studies have demonstrated that free-swimming infective larvae of multi-host parasites may be actively or accidentally consumed in large numbers by aquatic organisms, including invertebrates, fishes, and amphibians (Schotthoefer et al. 2007, Thieltges et al. 2008; Kaplan et al. 2009; Orlofske et al. 2012). Where these predators are not suitable hosts for the parasites, their increased metabolic rate (and hence appetite) under warmer temperatures can present another mechanism influencing rates of parasite transmission under elevated thermal conditions (Goedknegt et al. 2015).

### Susceptibility of intermediate hosts to infective parasites

Changing environmental temperatures have a wide range of effects on the biology of prospective intermediate hosts that potentially play a key role in determining their availability and/or suitability to act as hosts for heteroxenous parasites. These may be conceptually divided into two types of effects; those that alter the probability that the infective parasites and susceptible hosts coexist spatiotemporally (i.e., the probability of exposure occurring), and those that affect the ability of hosts to resist infection following exposure to infectious agents (i.e., the probability of infection for any given level of exposure).

First, we examine mechanisms by which altered temperatures influence the probability of spatiotemporal overlap of host and parasite; i.e., the probability that exposure occurs. The natural habitat selection behaviors of free-living animals are shaped by a range of factors that include the physicochemical environment as well as the availability of prey, shelter, mates, and predators (Morris 2003). Therefore, any change in the distribution of these key ecosystem components will likely influence the distribution of prospective host taxa (Beaugrand et al. 2015), and hence their availability in space to infective parasite stages. Furthermore, for taxa that exhibit ontogenetic or body-size dependent shifts in habitat use, including many species of fish, altered growth rates associated with elevated temperatures might similarly reduce the likelihood that intermediate hosts will occupy the same habitat as host-seeking parasites with which they have coevolved (Pickles et al. 2013), and possibly expose them to novel infections. Altered thermal regimes may also change the timing of key life history events—including the timing of host migrations, or of the reproduction, multiplication, or development of hosts or parasites—generating temporal mismatches between hosts and infective parasites (Hakalahti et al. 2006). For example, infective cercariae of the trematode *Ribeiroia ondatrae*, a parasite responsible for limb malformations in some amphibian species, are released by infected snails several months earlier under water temperatures elevated experimentally by 3 °C, and far in advance of the emergence of susceptible tadpoles (Paull and Johnson 2014).

The probability of hosts being exposed to aquatic parasites might be influenced in other ways by altered behavior allied to changing thermal regimes. Essentially any aspect of temperature-dependent behavior can serve to alter the spatiotemporal relationships of hosts and parasites and hence affect the likelihood of encounter. For example, the effects of increasing temperature on the metabolic rate of ectothermic organisms can influence the rate of food consumption (Roessig et al. 2005), potentially driving a greater exposure to trophically-transmitted parasites, either because prospective hosts eat more prey items, or because they become less selective in prey choice with increasing appetite. Similarly, increased rates of ventilation associated with increases in metabolic rate and reduced oxygen saturation at higher temperatures (Burton 1979) lead to increased levels of parasite infections that infect via the gills (Mikheev et al. 2014) and may generate stronger plumes, which can be tracked by motile parasites.

Second, we examine mechanisms that affect the probability of successful invasion of intermediate hosts following exposure. Perhaps the most obvious way in which the susceptibility of prospective hosts is affected by changing environmental temperatures is through associated shifts in the efficacy of the immune response. Host immune defences can be regarded as a key physiological barrier against parasite invasion (Leicht and Seppälä 2014). Physical contact between invading parasites and prospective hosts generally elicits a stimulation of the prospective host’s immune system (Wakelin 1996). While there are important differences between the immune systems of the different taxa of aquatic organisms acting as hosts for multi-host parasites, temperature plays a key role in determining the efficacy of immune responses of both aquatic invertebrates (Seppälä and Jokela 2011) and vertebrates (Le Morvan et al. 1998; Ellis 2001; Bowden 2008). Altered thermal regimes are, therefore, likely to have important consequences for the outcome of parasite exposures. For example, the resistance of the gastropod *Lymnaea stagnalis* invading trematode parasites is reduced at higher temperatures (Leicht et al. 2013; Paull et al. 2015). The efficacy of some components of the gastropod immune system is affected by high temperatures (30 °C) experienced during heat waves, potentially decreasing resistance to these parasites (Seppälä and Jokela 2011). At such high temperatures, the phenoloxidase activity and antibacterial activity of the snails is reduced but haemocyte concentration is maintained, suggesting that not all elements of the invertebrate immune response are equally affected, and raising the possibility that other types of infections may be affected differently (Seppälä and Jokela 2011).

Changing thermal regimes also have far-reaching implications for the susceptibility of ectothermic vertebrate hosts to parasite infections. In an experimental study of the effects of an experimentally-induced heat wave on two populations of three-spined stickleback fish, Dittmar et al. (2014) examined the implications of extreme, high temperature for immune performance and recovery. The authors expected the immune system to be less active at low temperatures, and even more suppressed at high temperatures. However, both innate and adaptive immune activity levels were found to be highest at lower temperatures (13 °C), with exposure to the simulated heat wave inducing long-lasting immune disorders that were predicted to have persistent consequences for subsequent immunocompetence and parasite resistance. As heat waves are expected to increase in both frequency and severity under altered climate scenarios (Meehl and Tebaldi 2004), developing a better understanding of the implications for host–parasite interactions appears to be critical.

### Development of parasites in intermediate hosts

The effect of environmental temperature on the rate at which larval stages of multi-host parasites develop within the bodies of their hosts is a potentially important factor in determining the dynamics of life cycles. One group of aquatic parasites for which the impacts of temperature on the rate of development has been studied in detail is the trematodes, which undergo asexual multiplication in the first intermediate (gastropod) host. Increased environmental temperatures are typically associated with a dramatic increase in the output of cercariae into the external environment, since higher temperatures both accelerate the production of cercariae within developing rediae and trigger the emergence of cercariae (Ataev 1991). However, while most studies have documented strong positive effects of elevated temperatures on mean cercarial output (Evans 1985; Shostak and Esch 1990; Paull et al. 2015), these effects are not universal. Some species appear to show reversed trends (Koprivnikar and Poulin 2009) and there is also evidence that such effects may sometimes be temporary (Paull et al. 2015), and variable between genotypes of a single parasite species (Berkhout et al. 2014). Additionally, any temporary increase in the release of cercariae under elevated temperatures could be offset by a shorter suitable season, possibly even leading to lower overall output of cercariae (de Montaudouin et al. 2015). A comparative analysis by Poulin (2006) suggested that the most marked positive effects of elevated temperature on cercarial release are found among species from more temperate latitudes, which are also those most likely to experience highest thermal shifts.

The effects of temperature on the development of metacercariae of the intertidal trematode *Maritrema novaezealandensis* in the amphipod *Paracalliope novizealandiae* have also been studied experimentally. The temperature at which amphipods were kept strongly affected the development stage of metacercariae recovered at 12 days post-infection; those from hosts kept at 15°C and 20 °C comprised almost entirely non-infective immature and early cyst stages, whereas at 25 °C almost a third had completed their development and were infective to the next host. At higher temperatures, amphipod survival was substantially reduced, curtailing parasite development. Increasing temperatures can, therefore, enhance parasite development, as long as this temperature can be tolerated by the host (Studer et al. 2010).

Outside the Trematoda, less is known about the consequences of elevated temperatures for rates of development of parasite larvae in aquatic hosts, although studies of acanthocephalan and nematode parasites of terrestrial animals suggest that such mechanisms are likely to be important (Halvorsen and Skorping 1982; Moore and Freehling 2002). Since most larval stages of heteroxenous parasites require a minimum period of growth and development in intermediate hosts to become competent to establish in subsequent hosts, their rate of development has the potential to influence life cycle dynamics. Faster development of larvae under warmer temperatures has been demonstrated experimentally in *Angiostrongylus cantonensis*, a nematode parasite of humans and the causative agent of eosinophilic meningitis, which uses the freshwater snail larvae *Pomacea canaliculata* as its first intermediate host. Larval nematodes complete the transformation from first stage larvae to human-infective third stage larvae in a shorter time (ca. 20 days) at 28 °C compared to 19 °C, at which completion takes more than two months (Lv et al. 2006). Similarly, the development rate of the acanthocephalan *Polymorphus marilis* in laboratory-infected *Gammarus lacustris* increased linearly with temperatures between 10°C and 25 °C (Tokeson and Holmes 1982). Faster larval development rates have implications for the number of life cycles that could be completed per year, and consequently the number of hosts that are likely to be exposed to the parasite.

As well as reducing the time taken for a life cycle to be completed, increasing the development rate of larval stages also shortens the period of time that parasites are at risk of pre-transmission host mortality (e.g., through predation), as well as promoting the onset of parasite-induced manipulation of host behavior (Moore 2002; Poulin 2010), either of which could increase the efficiency of transmission. Plerocercoids of the diphyllobothriidean cestode *Schistocephalus solidus* infect three-spined sticklebacks *Gasterosteus aculeatus*, growing to a large size in the body cavity of infected fish. The stickleback-*Schistocephalus* host–parasite system is an established model for the experimental study of host–parasite interactions (Barber and Scharsack 2010; Barber 2013). Experimental infection studies have demonstrated that the size attained by plerocercoids not only plays a key role in determining the timing of behavioral manipulation (Barber et al. 2004), but also influences the infectivity to subsequent hosts, the reproductive output of adults in the definitive host, and the energetic (Barber et al. 2008) and developmental consequences for both males and female hosts (Heins and Baker 2008; Macnab et al. 2009). Plerocercoid growth is also strongly influenced by environmental temperatures. In an experimental infection study, Macnab and Barber (2012) showed that, after 8 weeks, plerocercoids growing inside fish held at 20 °C had attained a mass of four times that achieved in fish held at 15 °C. Additionally, all of the plerocercoids recovered from fish held at the higher temperature, and none of those from the lower temperature, exceeded the 50 mg threshold for infectivity to the final host, a fish-eating bird. Elevated temperatures, therefore, have the potential to increase the rate at which infective parasite stages are transmitted to definitive hosts.

### Ecology and behavior of infected intermediate hosts

Thermal regimes can also interact with the behavior of host organisms to generate infection phenotypes that influence the dynamics of parasite life cycles (Labaude et al. 2015). For example, in some instances, infected hosts are known to select thermal regimes that negatively impact the development of their parasites (e.g., behavioral fevers/behavioral chills; Moore 2002; Moore and Freehling 2002), and/or aid recovery from parasitic infections. Such thermal preferences are regarded as host adaptations to manage parasite infections, and changing thermal regimes have the potential to impact the capacity of hosts to adopt such behaviors and create opportunities for either parasites or their hosts to benefit as a result.

Alternatively, parasites might adaptively manipulate the thermal preferences of their hosts and hence benefit from increased performance. When experimentally infected with the trematode *M. novaezealandensis*, the intertidal mollusc *Zeacumantus subcarinatus* exhibited a behavioral preference for higher temperatures (up to 40 °C), and also sustained activity for longer in warmer water before the onset of thermally-induced coma (Bates et al. 2011). In contrast to infections with a related parasite that did not induce these effects, development of *M. novaezealandensis* was heat tolerant and so the parasite potentially benefitted from the altered thermal preferences of its molluscan host. Similarly, three-spined sticklebacks infected with *S. solidus* exhibited preferences for warmer temperatures—which benefit plerocercoid growth but impair host performance—once the parasites they harboured exceeded the 50 mg infectivity threshold (Macnab and Barber 2012). A parsimonious explanation for these results is that altered thermal preferences represent adaptive manipulation by the parasite. Thermally altered environments might, therefore, provide greater opportunities for host-manipulating parasites to exploit host behavior. Conversely, parasite infection might trigger behavioral changes that increase host susceptibility to additional parasite infections, which at higher temperatures could increase host mortality (Mouritsen and Jensen 1997).

More generally, altered thermal environments that affect the developmental rates of manipulative parasites (section “Development of parasites in intermediate hosts”) have considerable potential to influence the patterns of parasite-induced behavior change that are observed, and where these behaviors influence transmission between successive hosts, life cycle dynamics will inevitably be affected (Labaude et al. 2015). Developing a better understanding of how changing environments—including altered thermal regimes—impact the behavioral interactions of hosts should be an important goal of future studies.

Extreme thermal warming, resulting from heat waves or unregulated thermal effluents, may disproportionately affect the mortality risk of hosts already infected with parasites. Wegner et al. (2008) studied patterns of mortality among three-spined stickleback families that were being maintained in an experiment during the 2003 European heat wave. During the experiment 78% of fish died, with the most heavily parasitized individuals suffering the highest mortality. Many of the parasites infecting these fish were pre-adult stages using sticklebacks as an intermediate host. Notwithstanding the possibility that scavengers (or predators) might become infected after ingesting dead (or moribund) fish, mass host die-offs during such events have clear potential to temporarily remove large numbers of infective parasite stages from aquatic ecosystems, with potentially severe implications for life cycle dynamics.

### Effects of changing thermal regimes on definitive hosts

Changes in environmental temperature are also likely to affect the dynamics of parasite life cycles through effects on the biology of definitive hosts. Host–parasite interactions in definitive hosts could be affected in several ways, most prominently through changes in host metabolism and immune response, diet composition, and distribution patterns. Many of the physiological effects of changing environmental temperatures that impact the biology and/or survival of ectotherm intermediate hosts (i.e., fish, reptiles, and amphibians) that have been discussed in sections “Development of parasites in intermediate hosts” and “Ecology and behavior of infected intermediate hosts” will also impact these organisms when they act as definitive hosts, and so will not be repeated. In contrast, for endotherm definitive hosts (i.e., birds and mammals), the metabolic rate is not expected to be directly affected by small temperature changes that fall within the thermoneutral zone (Buckley et al. 2012). However, temperature rises above the thermoneutral zone can reduce host metabolic rates, potentially leaving more energy to fuel immune responses (Morley and Lewis 2014), with possible negative consequences for parasites. Reduced metabolic rates of endotherms under elevated temperatures potentially reduce exposure to trophically acquired parasites if food consumption decreases. Alternatively, host diet composition may change as a result of altered food availability or host distribution (Thomas and Lennon 1999) arising from host phenology (Both et al. 2009). This can expose definitive hosts to a different set of parasites (Marcogliese 2002), and change the transmission of parasites between host stages (Marcogliese 2001). Changes in distribution are expected to be most prominent in definitive hosts, because this host stage typically exhibits the greatest vagility (Criscione and Blouin 2004). Moreover, altered host distribution can change the spatiotemporal distribution of parasites, including spread to new areas and presence at different times of the year, which can then facilitate host-switching (Brooks and Hoberg 2007). Overall, it is thus quite possible that more extreme temperature will increase the distribution and abundance of some parasites, through changes in definitive host phenology or distribution, while other species might go (locally) extinct, because of phenological mismatches with host life histories or patterns of migration.

### Effect on parasite sexual reproduction in the definitive host

The reproductive output of multi-host parasites infecting their definitive hosts may be influenced by environmental changes through two major routes. First, adult parasites, including those that occupy the gastrointestinal tract of definitive hosts, may be susceptible to host immune responses and/or the level of host nutrition that can be acquired, and either of these might be affected by thermal regimes. For parasites that undertake substantial growth and live for an extended period in the definitive host (e.g., many tapeworms) the level of host alimentation might affect adult growth and performance in much the same way as discussed in section “Development of parasites in intermediate hosts.”

Second, altered thermal regimes may impact the performance of adult parasites even if conditions in the definitive hosts are buffered from the effects of environments, if effects on earlier developmental stages incur “carryover” costs (O'Connor et al. 2014). There is widespread recognition of the importance of such phenomena in free-living animals, including holometabolous insects, amphibians, fish, and birds. For example, exposing the eggs of Asian lady beetles, *Harmonia axyridis*, to elevated temperatures during development had negative carryover effects on the performance of subsequent stages, which exhibited a reduction in longevity, oviposition period, and reproductive output of adults, while the pre-oviposition period became longer as the temperature increased (Zhang et al. 2014). There is also evidence that carryover effects are important in parasitoids. Individual *Aphidius colemani* parasitoid wasps forced through a shorter larval developmental period suffered decreased adult survival due to small lipid reserves, and decreasing lifetime reproductive output (Colinet et al. 2007). However, we are not aware of similar literature for multi-host parasites. Assuming that physiological and cellular processes do not differ fundamentally between parasitic and non-parasitic metazoan taxa, we would expect similar negative consequences for adult stages of parasites that have been exposed to sub-optimal developmental conditions. This appears to be a potentially suitable area for further investigation using experimentally amenable parasite systems.

## Conclusions, predictions, and directions for future research

Both the mean temperature and the incidence of extreme temperature events are expected to increase with predicted climate changes over the next century (Meehl and Tebaldi 2004; IPCC 2013). Predicting the consequences of these for the transmission of multi-host parasites in aquatic ecosystems will be enormously challenging because of the many ways in which temperature changes the biology of hosts, parasites, and the interactions between them. Also, the mean effects of temperature on individuals within a population might not reflect consistent underlying variation in host or parasite populations (Berkhout et al. 2014). This variation could lead to the selection of certain genotypes within populations. Furthermore, changes in temperature should not be considered in isolation, as other environmental changes might act synergistically or antagonistically (Marcogliese 2016). Nevertheless, the results of experimental studies using amenable host–parasite systems can give insights into the types of consequences that might arise. In general, negative effects appear to be most likely when extreme temperatures are encountered, whereas the consequences of incremental changes are less clear. One of the most likely outcomes of altered thermal regimes is a change in the spatiotemporal distribution of infective parasites—as a result of the altered timing of their release and of the number of infective stages released at the same time—rather than in the total output of infective parasite stages. This could mean that hosts may face greater spatiotemporal heterogeneity in the density of infective stages they encounter, with negative effects on survival for hosts present during the peaks of parasite release (e.g., Mouritsen and Jensen 1997; Meissner and Bick 1999). Another general prediction is that parasites with more complex life cycles, and especially those that exhibit high levels of specificity at one or more host stages, may be less likely to persist in altered climates, because of increasing uncertainty that new climates will suit all hosts and/or allow all required inter-host interactions to persist at the right time.

We have highlighted a number of knowledge gaps and other areas that might benefit from further investigation throughout our review, and briefly mention three topics of interest here. First, there is a lack of knowledge about how the effects of altered thermal regimes on developmental stages of multi-host parasites might impact the reproductive performance of adult forms. It seems entirely possible that even when all hosts are present and ecological interactions allow the completion of parasite life cycles, cumulative effects of physiological and developmental stress experienced throughout the parasite life cycle might generate carryover effects that impact on parasite fitness. Second, the critical role of physiological acclimation—of both hosts and parasites—on the outcome of host–parasite interactions under changing environmental regimes has received scant interest until very recently (de Montaudouin et al. 2015, Paull et al. 2015). It is unusual for thermal changes to occur instantaneously in aquatic ecosystems, and organisms will more often be exposed to gradually increasing temperatures. Fish, for example, are capable of substantial physiological changes, including to the immune response, during thermal acclimation (Podrabsky and Somero 2004; Marnila and Lilius 2015). The effects of acclimatization on parasites themselves, however, and the consequences of different rates of thermal change on the dynamics of interactions between aquatic hosts and heteroxenous parasites, remain largely unexplored. However, discrepancies between model predictions and field data show that parasite load is not a direct function of temperature (de Montaudouin et al. 2015) and thermal adaptation is a potentially key area for future investigation. Finally, we highlight the importance of interactions between parasite-mediated host behavioral change and changing thermal regimes as a potential area for further research. Although few studies have examined thermal preferences of infected hosts in detail, where hosts and parasites have different thermal optima, there is the potential for either party to use behavioral mechanisms to gain an advantage over the other. More generally, the frequency, severity, and ecological consequences of parasite-induced behavioral changes might all be mediated by changing environmental conditions, with far-reaching implications for the transmission of multi-host parasites and the ecology of their life cycles (Labaude et al. 2015).

While we have restricted ourselves in this review to considering the effects of changing temperatures *per se* on host–parasite interactions, a wide range of secondary environmental consequences of elevated temperatures that we do not address—including changes in water salinity, pH, and flow regimes in fluvial ecosystems—are also expected to influence host–parasite interactions. Furthermore, a number of unexpected mechanisms by which altered thermal regimes might influence patterns of infection have recently emerged, including a reduced capacity of thermally-challenged parasites to withstand infections by hyperparasites (Kaunisto et al. 2015). We hope that our review will stimulate researchers in this rapidly advancing field to consider these and other types of mechanisms that may play important roles.
